# Effectiveness of soft robotic glove versus repetitive transcranial magnetic stimulation in post-stroke patients with severe upper limb dysfunction: A randomised controlled trial

**DOI:** 10.3389/fneur.2022.887205

**Published:** 2023-01-11

**Authors:** Taotao Wang, Zhonghua Liu, Jianxiong Gu, Jizhi Tan, Tian Hu

**Affiliations:** ^1^Zhongshan People's Hospital, Zhongshan, China; ^2^Affiliated Hospital of Guangdong Medical University, Zhanjiang, China; ^3^Guangdong Medical University, Zhanjiang, China

**Keywords:** repetitive transcranial magnetic stimulation, soft robotic glove, motor dysfunction after stroke, central intervention, peripheral intervention

## Abstract

**Purpose:**

To explore the difference in rehabilitation effect between soft robot gloves and repetitive transcranial magnetic stimulation (rTMS) in patients with severe upper limb motor dysfunction after a stroke.

**Methods:**

A total of 69 post-stroke patients with severe upper limb dysfunction were randomly assigned to a repetitive transcranial magnetic group, a soft robotic glove group, and a conventional treatment group. The primary outcomes were the Fugl-Meyer Upper Extremity Assessment (FMA-UE) and the Modified Barthel Index (MBI). The secondary endpoints were the amplitude surface electromyogram of the extensor wrist muscle (sEMG) and the cerebral hemispheric resting motor threshold (RMT).

**Results:**

The change of FMA-UE score in the soft robotic glove group was significantly better than that in the conventional treatment group (median difference: 2 points; 95% confidence interval [1, 3]; *P* < 0.05), but there was no significant difference compared with the repetitive transcranial magnetic stimulation group (median difference: 0 points; 95% confidence interval [−1, 2]; *P* [0.547] > 0.05). There was no significant difference in the change of MBI score between the soft robotic glove group and the conventional treatment and repetitive transcranial magnetic treatment groups [F = 2.458, *P* [0.093] > 0.05]. There was no significant difference in the change of sEMG score between the soft robotic glove group and the conventional treatment and repetitive transcranial magnetic treatment groups [H = 0.042, *P* [0.980] > 0.05]. Additionally, the change of RMT score in the soft robotic glove group was significantly inferior to that in the repetitive transcranial magnetic treatment group [difference: −1.09; 95% confidence interval [−2.048, 0.048]; *P* < 0.05], but there was no significant difference compared with the conventional treatment group [difference: 0.31 points; 95% confidence interval [−0.879, 0.358]; *P* [0.495] > 0.05].

**Conclusion:**

For patients with severe dyskinesia after a stroke, soft robotic gloves are as effective as repetitive transcranial magnetic stimulation and may be a good choice for home rehabilitation. In addition, conventional treatment combined with repetitive transcranial magnetic stimulation (rTMS) or a soft robotic glove produced better rehabilitation outcomes than conventional treatment alone.

## Introduction

Over the past 20 years, great progress has been made in the treatment and prevention of stroke, and the mortality rate of stroke has decreased; however, the disability rate caused by stroke disease remains high (1, 2). In order to reduce the disability rate and minimize the burden on families and national health systems, patients after stroke need to receive the most appropriate and effective rehabilitation interventions in a timely (3). At present, how to choose rehabilitation intervention protocol for patients with upper limb motor dysfunction after stroke and which one can get the best rehabilitation effect has become the focus of current research in the field of stroke rehabilitation (4).

It is well known that stroke patients are often left with motor, cognitive, speech, psychological, and other functional disorders, among which upper limb motor dysfunction is one of the most common complications and the main reason for preventing patients from achieving basic self-care and reducing their quality of life (5). The rehabilitation process of upper limb motor function after a stroke is long and the efficacy is not significant, especially for severe upper limb motor dysfunction. Although there are an increasing number of studies investigating the rehabilitation of upper limb motor dysfunction after a stroke, there are also more and more related interventions (6). However, for patients who have severe upper limb motor dysfunction, the rehabilitation process is lengthier, there are fewer therapeutic possibilities, the effect is not immediately apparent, and the prognosis is poor. Therefore, the rehabilitation of severe upper limb motor dysfunction is a daunting challenge for practitioners (7).

At present, rehabilitation interventions for upper limb motor dysfunction can be divided into central intervention and peripheral intervention (8). Central intervention mainly refers to the direct stimulation of the motor cortex of the brain, which controls the movement of the upper limb and improves motor function by changing the excitability of the corresponding central motor cortex (9). In recent years, repetitive transcranial magnetic stimulation (rTMS), a central rehabilitation intervention method for directly stimulating the motor cortex of the brain, has attracted the attention of many researchers (10). Studies have found that rTMS can rebalance interhemispheric inhibition by upregulating or downregulating the excitability of the cerebral cortex, thus achieving the effect of improving the corresponding motor dysfunction (11). This method has been applied to the clinical treatment of upper limb motor dysfunction after a stroke in many large grade A hospitals in China and has received good feedback (12). However, owing to the high price of transcranial magnetic equipment and the high charge for each treatment, it has not been widely promoted in primary and community hospitals. Peripheral intervention mainly refers to the direct stimulation of the affected limb, which is fed back to the brain center through repeated external interventions, such as movement or proprioception, so as to promote the remodeling of brain and nerve function (8, 13). Brochards et al.'s study showed peripheral interventions such as robotics rehabilitation, can provide repetitive movements of limbs to effectively improve upper limb movement disorders after stroke (14). And soft glove robotic as a device in peripheral intervention, soft glove robotic can make up for the deficiency of the artificial, increase the intensity of treatment at a very affordable cost and provide advantages such as precise and controllable assistance or resistance during exercise, good repeatability, and improved patient motivation for training (15). Owing to their small size, ease of movement, and appropriate price, soft robotic gloves have a large audience in primary hospitals and community rehabilitation institutions, and some patients even buy them for use at home (16).

In fact, in clinical work, we know that patients with severe upper limb motor dysfunction cannot be treated in large grade A hospitals for a long time due to the long treatment cycle and high cost; family rehabilitation is their final choice (17). Currently, many central intervention treatment devices are mainly concentrated in large hospitals and are unable to be used in the home. Some small items of peripheral intervention rehabilitation training equipment can not only allow patients to achieve enough exercise but are also more convenient to use in the community and at home. For patients with severe upper limb motor dysfunction, there is no substantial evidence as to whether one intervention is better or whether the outcomes of these interventions differ significantly.

The main objective of this study was to observe whether there was a significant difference in rehabilitation effect between conventional treatment combined with repetitive transcranial magnetic stimulation (central intervention) or soft machine gloves (peripheral intervention) in patients with severe upper limb motor dysfunction after a stroke. In addition, it can help determine whether soft robotic gloves (which can be used at home) can be used as an alternative option in the absence of repeated TMS (used in large hospitals).

## Materials and methods

The study was approved by the Ethics Committee of the Affiliated Hospital of Guangdong Medical University (acceptance number, 2020-052-01) and registered with the China Clinical Trial Center (Registration number, ChiCTR2000037959). The purpose of this study and the related ethical issues were communicated to the patients and informed consent was obtained. The researchers promised that the patient data obtained in this study would be kept properly and would only be used only for academic research.

### Participants

Sixty-nine hemiparetic stroke patients were enrolled at the Rehabilitation Medicine Department of The Affiliated Hospital of Guangdong Hospital from June 2020 to June 2021. In this study, the test level α = 0.05, the test power 1-β = 0.8, and the score of the FMA-UE scale was used as the effective index. According to previously published literature (18–20), the mean effect of conventional treatment was 6.89 and the standard deviation was 0.09; the mean effect of conventional therapy combined with repetitive transcranial magnetic stimulation was 14.9 and the standard deviation was 4.4; and the mean effect and standard deviation of conventional treatment combined with rehabilitation robot treatment were 11.5 and 1.7, respectively. Calculations using PASS15.0 software suggested that the required sample size for each group was 21. All patients met the following inclusion criteria: (i) stroke was confirmed by transcranial CT or MRI examination; (ii) clear consciousness and good cooperation, defined by scores above 21 in the mini-mental state examination; (iii) severe paresis of an upper limb, as defined by Brunnstrom approach stage ≤ 3 and a Fugl–Meyer Assessment score below 50; (iv) aged 8–80 years old; and (v) agreed to sign the informed consent form. The exclusion criteria were as follows: (i) serious heart, lung, liver, kidney, and other important organ diseases; (ii) in addition to upper limb motor dysfunction caused by a stroke, there were other contributing factors (such as fractures, skin trauma, nerve damage, etc.); (iii) a history of epilepsy; and (iv) presence of metal implants in the brain or neck, pacemakers, cochlear implants, etc.

### Clinical procedure

**Figure 1:**
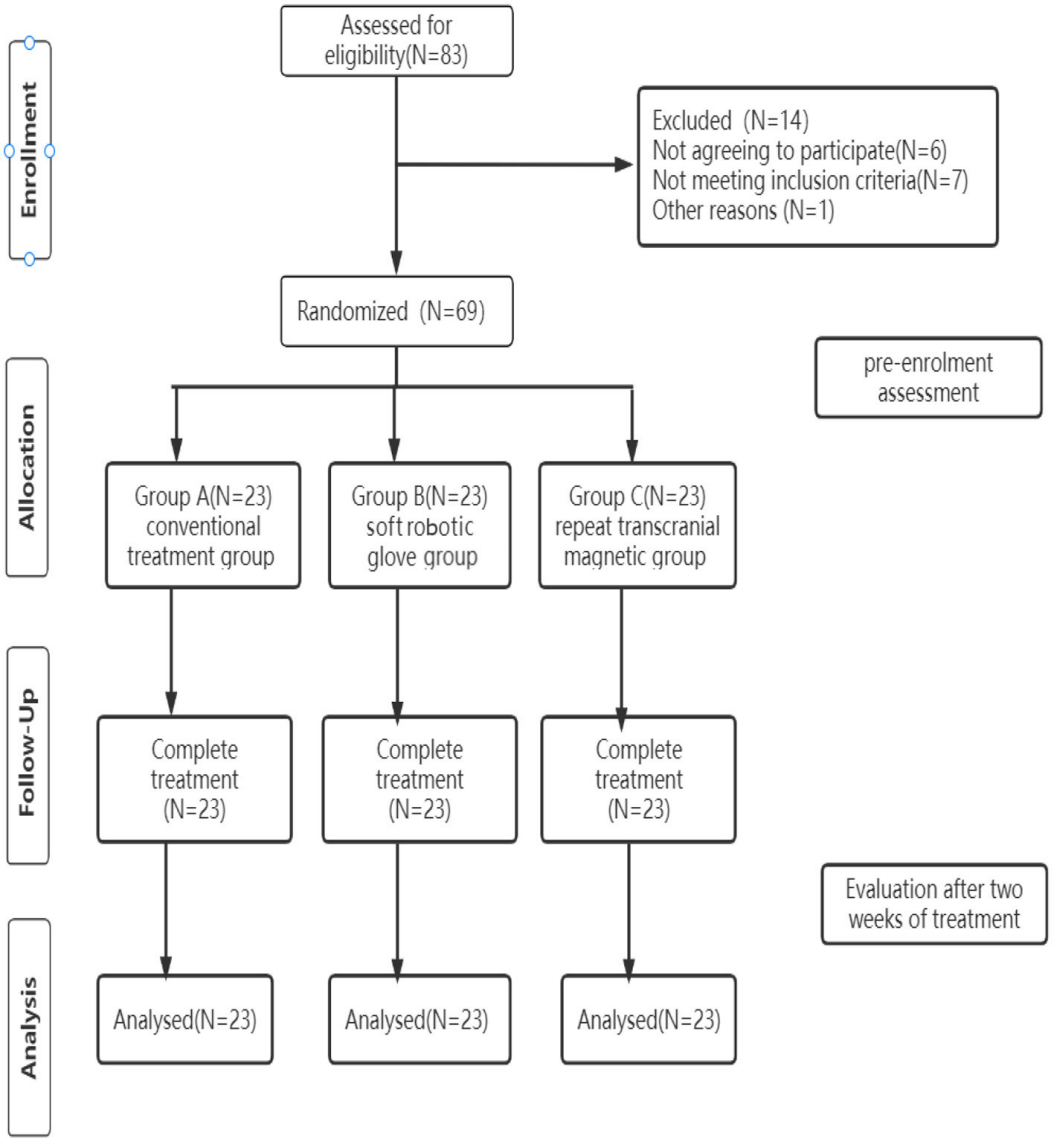
Flowchart of the experimental design.

A flowchart of the study design is shown in Figure 1.

This study was a single-center randomized controlled prospective clinical trial. Sixty-nine patients, who were strictly screened according to inclusion and exclusion criteria, were randomly divided into a conventional treatment group, a soft robotic glove group, and a repetitive transcranial magnetic group. Randomization order was computer generated and concealed in sequentially numbered opaque envelopes. The clinical demographic characteristics of the participants are listed in Table 1.

**Table 1:** Demographic and clinical characteristics.

	**Group A** ***N* = 23**	**Group B** ***N* = 23**	**Group C** ***N* = 23**	***P***
Sex (M/F)	17/6	12/11	16/7	0.261^a^
Age (year)^#^	61.78 ± 12.63	61.78 ± 11.37	62.22 ± 9.65	0.989^c^
Onset (Days)^*^	90.52 ± 17.47	92.30 ± 16.77	101.96 ± 21.66	0.898^c^
Affected side (L/R)	9/14	11/12	14/9	0.332^a^
Stroke (I/H)	11/12	9/14	9/14	0.788^a^
Lesion location				0.735^a^
Cortical	11	10	10	
Subcortical	8	8	11	
Both	4	5	2	
FMA-UE^*^	14 (16)	9 (12)	11 (8)	0.494^b^
MBI^#^	47.09 ± 14.52	50.17 ± 14.45	51.17 ± 13.90	0.602^c^
ΔsEMG^*^	16 (94)	12 (17)	14 (30)	0.688^b^
RMT^#^	31.09 ± 7.31	32.52 ± 7.73	30.00 ± 8.54	0.556^c^

Group A, conventional treatment group; group B, soft robotic glove group; group C, repetitive transcranial magnetic group; M, male; F, female; L, left; R, right; I, ischemic; H, hemorrhagic.

^*^Data are Median (quartile spacing); ^#^Data are mean ± SD; P^a^, chi-squared test; P^b^, Kruskal–Wallis H test; P^c^, one-way ANOVA; FMA-UE, Fugl–Meyer assessment of upper extremity; MBI, Modified Barthel Index; RMT, resting motor threshold; ΔsEMG, amplitude of sEMG of extensor wrist muscle.

### Intervention

The conventional treatment group used a conventional rehabilitation treatment program, the soft robotic glove group used a conventional rehabilitation program combined with soft robotic glove treatment, and the repetitive transcranial magnetic group used a conventional rehabilitation program combined with repetitive transcranial magnetic stimulation treatment. The three groups of subjects all received the corresponding rehabilitation treatment once a day for 2 consecutive weeks. The rehabilitation intervention program of the three groups was formulated according to the FITT principle.

### The conventional rehabilitation treatment group

**Figure 2:**
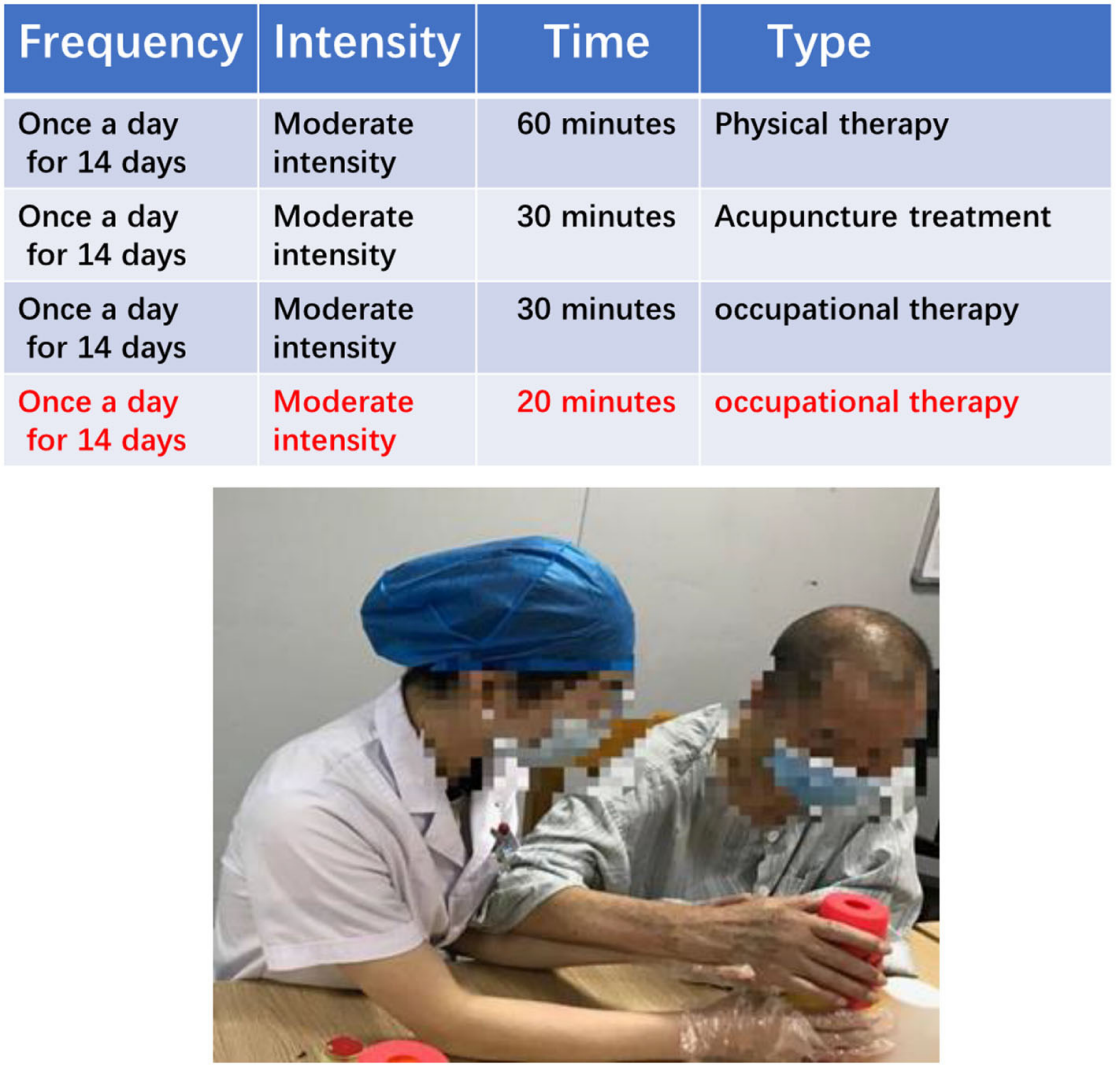
A rehabilitation therapist and patient in one-on-one training.

The conventional treatment group rehabilitation scheme is based on the principles of FITT and primarily consisted of physical therapy, acupuncture, and occupational therapy (Figure 2). In addition, 20 min were added after the regular 30 min of occupational therapy.

### The soft robotic glove treatment group

**Figure 3:**
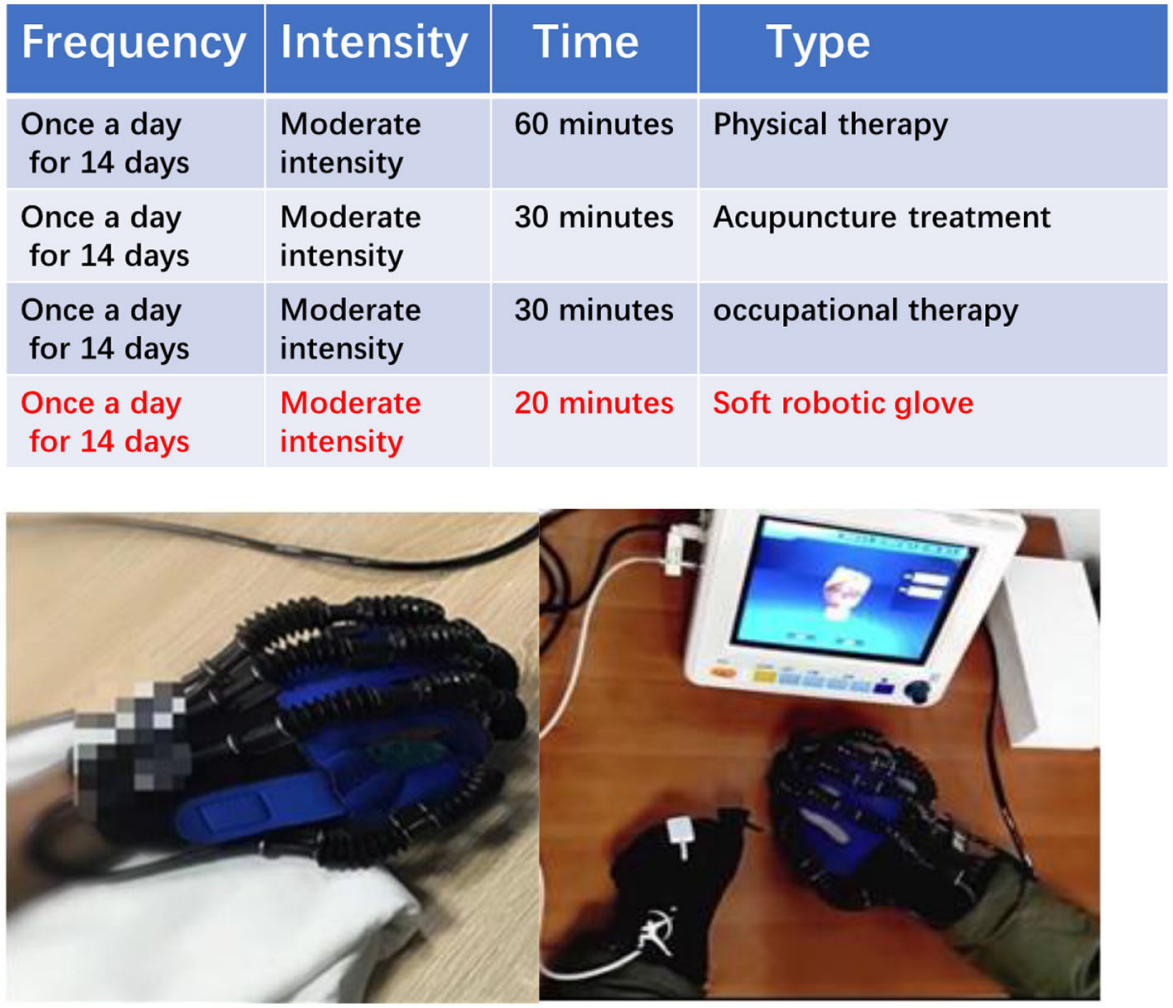
A patient wearing soft robotic gloves for treatment.

The soft robotic glove group rehabilitation scheme is based on the principles of FITT, and primarily consists of physical therapy, acupuncture, and occupational therapy. In addition, 20 min of soft robotic glove training were added after the regular 30 min of occupational therapy. The soft robotic glove training involved patients wearing pneumatic rehabilitation soft gloves and carrying out a back extension of the wrist and flexion and extension of the hand passively or with assistance (Figure 3).

### The repetitive transcranial magnetic stimulation group

**Figure 4:**
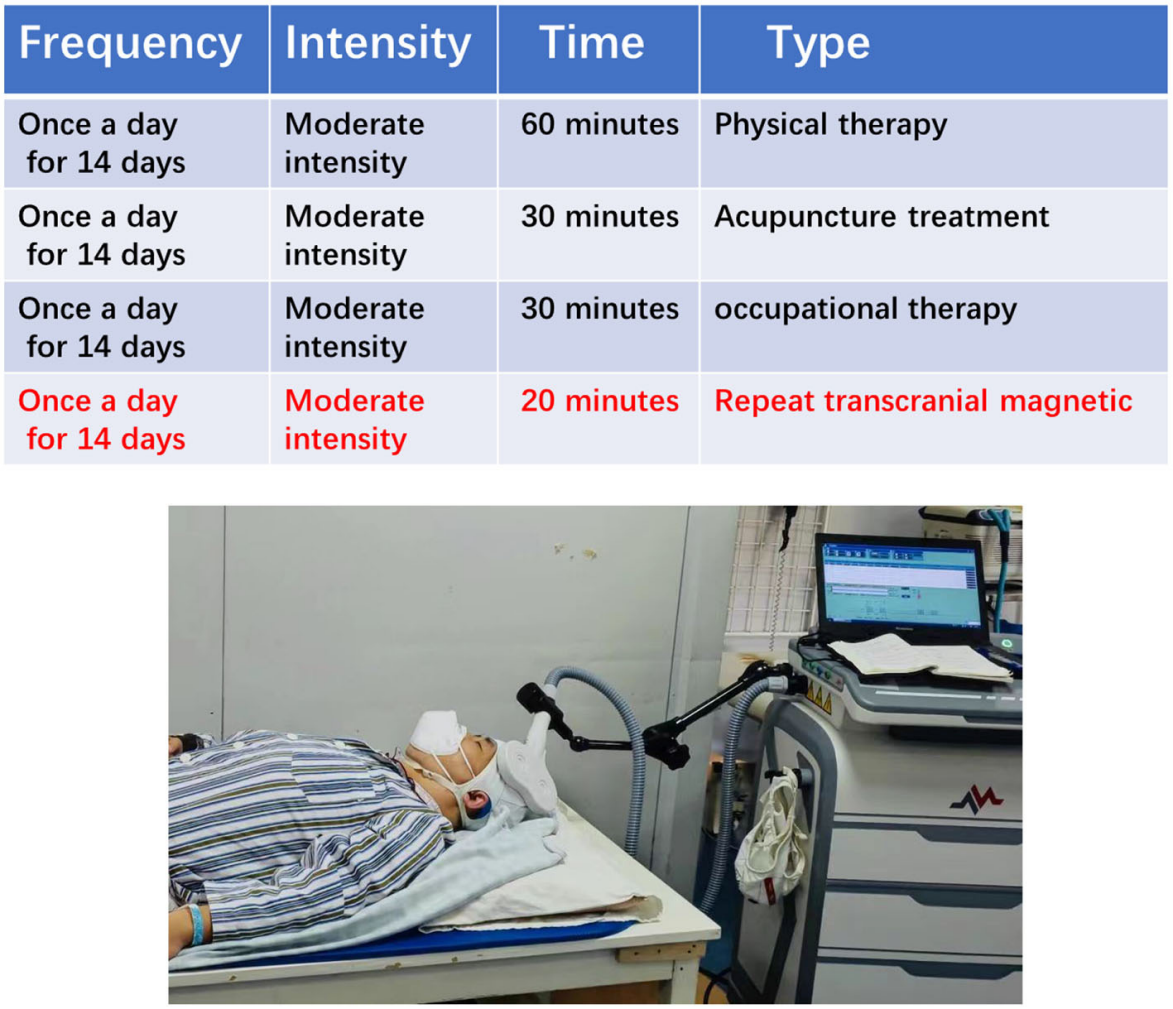
A patient undergoing repeat transcranial magnetic therapy.

The repeat transcranial magnetic group rehabilitation scheme is based on the principles of FITT and primarily consists of physical therapy, acupuncture, and occupational therapy. In addition, 20 min of repetitive transcranial magnetic treatment were added after the regular 30 min of occupational therapy. Repetitive transcranial magnetic treatment involved the patient lying in a quiet state with their head fixed, and 8-shaped coil was placed in the M1 area of the contralateral cerebral cortex. The treatment was carried out with an intensity of 90% RMT and a frequency of 1 HZ and consisted of stimulation for 10 s followed by a 2-s pause for 20 min every day during the treatment period (Figure 4).

### Outcome measures (Assessed before study inclusion and after 2 weeks of treatment)

**Figure 5:**
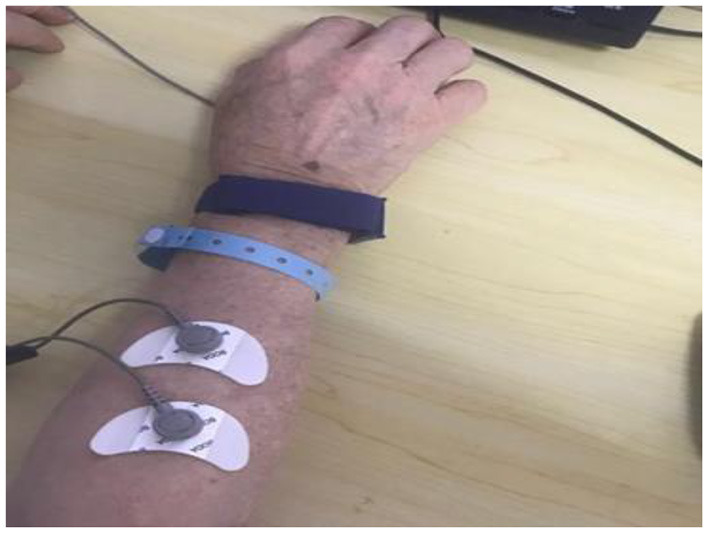
Measurement of surface electromyography of extensor carpi dorsi muscles.

**Figure 6:**
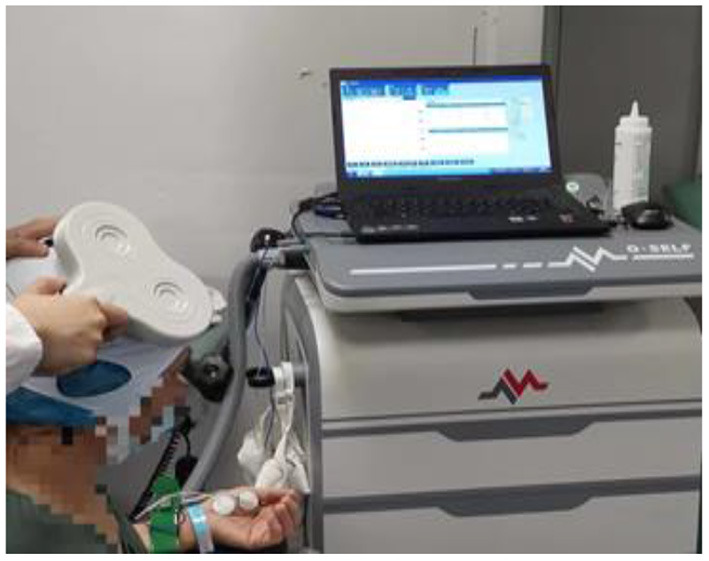
Measurement of the resting motor threshold of the cerebral cortex.

1. Fugl–Meyer Upper limb rating scale (FMA-UE) (21): includes 33 items of the upper limb and hand motor function (a total of 66 points), mainly reflecting the recovery of the upper limb and hand motor function; the higher the score, the better the motor function.
2. Modified Barthel Index (MBI) (22): includes 10 items, such as dressing, grooming, and walking, with a maximum score of 100. The index mainly reflects the patient's ability to carry out the basic tasks of daily living and self-care. The higher the score, the better the self-care ability and the higher the quality of life.
3. ΔsEMG (the difference of the potential amplitude of sEMG during exercise and rest) (23): the difference of the potential amplitude of the dorsal extensor muscle of the wrist during rest and exercise was measured using a Myo-ex surface myoelectric induction device in the E-LINK system, which mainly reflects the change in muscle strength of the dorsal extensor muscle group of the wrist (Figure 5).
4. RMT (resting motor threshold of the cerebral cortex) (24): while the patient was in a resting state, the recording electrode and reference electrode were fixed on the abductor pollicis brevis on the affected side, the grounding wire was clamped on the wrist, and the center of the coil was placed in the thumb position indicated by the positioning cap. The amplitude of MEP was >50V in five out of 10 single pulse stimuli. The measurement mainly reflects the changes in the excitability of the cerebral cortex (Figure 6).

### Statistical analysis

SPSS22.0 was used for statistical analysis. A chi-square test was used for classified variables. Continuous variables were used to judge whether the data conformed to a normal distribution. Data that conformed to a normal distribution are expressed as mean ± standard deviation. Data that did not conform to a normal distribution are expressed as median (quartile interval), and a one-way ANOVA (consistent with normal distribution data) or a non-parametric Kruskal–Wallis test (non-normal distribution data) were used for comparisons between the three groups. A paired *t*-test (consistent with normal distribution data) or a Wilcoxon rank-sum test (non-normal distribution data) were used to compare the values of each group before and after the intervention. *P* < 0.05 was considered statistically significant.

## Results

### Participants' characteristics

A total of 69 patients completed the trial. There was no significant difference in the clinical demographic characteristics and baseline scores between the different groups. (*P* > 0.05) (Table 1).

#### Upper limb motor function

When comparing within groups, the FMA-UE scores of the three groups before and after treatment were statistically significant (*P* < 0.05). When comparing between groups, there was no significant difference in FMA-UE scores between the three groups before or after treatment (*P* > 0.05). The difference in the FMA-UE scores (improvement: change from baseline to post-intervention score) was significantly different among the three groups before and after treatment (*P* < 0.05) (Table 2). Further pairwise comparison between the three groups showed that there were significant differences between group A and group B (*P* < 0.05) and between group A and group C (*P* < 0.05) but no significant differences between group B and group C (*P* > 0.05) (Tables 3, 4).

**Table 2:** Comparison of FMA-UE scores before and after treatment among the three groups.

**Group**	T0 (FMA)^*^	T1 (FMA)^*^	ΔFMA^*^	***P***
Group A	14 (16)	15 (18)	2 (2)	**<0.001** ^**c**^
Group B	9 (12)	15 (10)	4 (2)	**<0.001** ^**c**^
Group C	11 (8)	15 (10)	4 (3)	**<0.001** ^**c**^
H	1.412	0.037	20.030	
P	0.494^d^	0.982^d^	**<0.001** ^d^	

Group A, conventional treatment group; group B, soft robotic glove group; group C, repetitive transcranial magnetic group; ^*^M (IQR), median (interquartile range); T0 (FMA), pre-treatment assessment; T1 (FMA), post-treatment assessment; ΔFMA, difference of the FMA-UE score before and after treatment; P^c^, statistic from a non-parametric test of relevant samples; P^d^, statistic from a non-parametric Kruskal–Wallish test.

**Table 3:** Pairwise comparison of the difference in FMA-UE scores between the three groups before and after treatment.

	**H**	***P***	***P* ^(A vs. B)^**	***P* ^(A vs. C)^**	***P* ^(C vs. B)^**
ΔFMA	20.030	**<0.001**	**<0.001**	**<0.001**	0.547

Group A, conventional treatment group; group B, soft robotic glove group; group C, repetitive transcranial magnetic group.

**Table 4:** Comparison of ΔFMA between the soft machine glove group and the conventional therapy and repetitive transcranial magnetic stimulation groups.

	**Group B vs. Group A**				**Group B vs. Group C**			

	**Group B**	**Group A**	**Median difference** (95% CI)^*^	***P***	**Group B**	**Group C**	**Median difference** (95% CI)^*^	***P***
ΔFMA	4 (2)	2 (2)	2 (1, 3)	**<0.001**	4 (2)	4 (3)	0 (−1, 2)	0.547

^*^Non-normally distributed data with a median 95% CI using the Hodges–Lehmann method; group A, conventional treatment group; group B, soft robotic glove group; group C, repetitive transcranial magnetic group; ΔFMA, difference in FMA-UE score before and after treatment.

#### Self-care ability in daily life

When comparing within groups, the MBI scores of the three groups before and after treatment were statistically significantly different (*P* < 0.05). When comparing between groups, there was no significant difference in MBI scores between the three groups before and after treatment (*P* > 0.05), and the difference in MBI scores before and after treatment (improvement: change in scores from baseline to post-intervention) was not significantly different among the three groups (*P* > 0.05) (Table 5).

**Table 5:** Comparison of MBI scores before and after treatment among the three groups.

**Group**	T0 (MBI)^*^	T1 (MBI)^*^	ΔMBI^*^	***P***
A	47.09 ± 14.52	60.35 ± 15.52	13.91 ± 6.25	**<0.001** ^e^
B	51.17 ± 13.90	61.52 ± 14.71	10.35 ± 4.70	**<0.001** ^e^
C	50.17 ± 14.45	61.22 ± 12.01	11.04 ± 6.26	**<0.001** ^e^
F	0.511	0.043	2.458	
P	0.602^b^	0.958^b^	0.093^b^	

Group A, conventional treatment group; group B, soft robotic glove group; group C, repetitive transcranial magnetic group; ^*^±SD, mean ± standard deviation of data conforming to a normal distribution; T0 (MBI), evaluation before treatment; T1 (MBI), evaluation after treatment; ΔMBI, difference in MBI score before and after treatment; P^b^, statistic from one-way ANOVA; P^e^, statistic from a paired t-test.

#### The amplitude of EMG of the extensor wrist muscle

When comparing within groups, the surface electromyographic amplitude (sEMG) of the wrist dorsal extensor muscle group before and after treatment in the three groups was statistically significantly different (*P* < 0.05). When comparing between groups, there was no significant difference among the three groups before and after treatment (*P* > 0.05), and the difference in the surface electromyography amplitude (sEMG) of the wrist dorsal extensor muscle group scores before and after treatment (improvement: change in scores from baseline to post-intervention) was not significantly different among the three groups (*P* > 0.05) (Table 6).

**Table 6:** Comparison of surface electromyographic amplitude (sEMG) of the wrist dorsal extensor muscle group before and after treatment among the three groups.

**Group**	T0 (sEMG)^*^	T1 (sEMG)^*^	ΔsEMG^*^	***P***
A	16 (24)	22 (32)	4 (12)	**<0.001** ^c^
B	14 (30)	23 (36)	5 (10)	**<0.001** ^c^
C	12 (17)	16 (23)	4 (8)	**<0.001** ^c^
H	0.688	0.386	0.042	
P	0.748^d^	0.824^d^	0.980^d^	

^*^M (IQR) median (interquartile range); T0 (sEMG), pre-treatment evaluation; T1 (sEMG), post-treatment evaluation; ΔsEMG, difference of surface EMG amplitude of wrist dorsal extensor muscle group before and after treatment; P^d^, statistic of the non-parametric Kruskal–Wallis H-test; Pc, statistic from a non-parametric test of relevant samples; group A, conventional treatment group; group B, soft robotic glove group; group C, repetitive transcranial magnetic group.

#### Resting motor threshold

When comparing within groups, there was no significant difference in the RMT before and after treatment in groups A and B (*P* > 0.05), but there was a statistically significant difference in group C before and after treatment (*P* < 0.05). When comparing between groups, there was no significant difference in RMT scores between the three groups before or after treatment (*P* > 0.05). The RMT score (improvement: change from baseline to post-intervention score) was significantly different among the three groups before and after treatment (*P* < 0.05) (Table 7). Further pairwise comparison between the three groups showed that there were significant differences between groups A and C (*P* < 0.05) and between groups B and C (*P* < 0.05), but no significant differences between groups A and B (*P* > 0.05) (Tables 8, 9).

**Table 7:** Comparison of RMT scores before and after treatment among the three groups.

**Group**	T0 (RMT)^*^	T1 (RMT)^*^	ΔRMT^*^	***P***
A	31.09 ± 7.31	31.52 ± 7.17	0.43 ± 0.11	0.086^e^
B	32.52 ± 7.73	33.04 ± 7.42	0.74 ± 0.14	0.143^e^
C	30.00 ± 8.54	33.52 ± 9.18	1.83 ± 0.22	**<0.001** ^e^
F	0.592	0.395	5.436	
P	0.556^b^	0.676^b^	**0.007** ^b^	

±SD, mean ± standard deviation of data conforming to a normal distribution; T0 (RMT), pre-treatment evaluation; T1 (RMT), post-treatment evaluation; ΔRMT, difference in RMT score before and after treatment; P^b^, statistic from one-way ANOVA; P^e^, statistic from a paired t-test. group A, conventional treatment group; group B, soft robotic glove group; group C, repetitive transcranial magnetic group.

**Table 8:** Pairwise comparison of RMT differences before and after treatment among three groups.

	**F**	**P**	***P* ^(A vs. B)^**	***P* ^(A vs. C)^**	***P* ^(B vs. C)^**
ΔRMT	5.436	0.007^c^	0.495	**0.003**	**0.017**

ΔRMT, difference in RMT score before and after treatment; P^c^, one-way ANOVA.

**Table 9:** Comparison of ΔRMT between the soft machine glove group and the conventional therapy and repetitive transcranial magnetic stimulation groups.

	**Group B vs. Group A**				**Group B vs. Group C**			

	**Group B**	**Group A**	**Mean difference**^#^ **(95% CI)**	***P***	**Group B**	**Group C**	**Mean difference**^#^ **(95% CI)**	***P***
ΔRMT	0.74 ± 0.14	0.43 ± 0.11	0.31 (−0.879, 0.358)	0.495	0.74 ± 0.14	1.83 ± 0.22	−1.09 (−2.048, 0.048)	**0.017**

^#^Normally distributed data (a t-test was used to calculate the mean difference with 95% CI); ΔRMT, difference in RMT scores before and after treatment; group A, conventional treatment group; group B, soft robotic glove group; group C, repetitive transcranial magnetic group.

## Discussion

This study investigated the effects of combined soft robotic gloves compared with combined repetitive transcranial magnetic stimulation and conventional therapy on upper limb function in stroke patients with severe hemiplegia. After 14 consecutive corresponding rehabilitation interventions, the combination of soft gloves was superior to conventional treatment for the recovery of motor function in patients with severe upper limb dysfunction post stroke. There was little difference between the combination of soft gloves and repeated transcranial magnetic therapy, and there was no significant difference between the three groups in terms of improving the ability to perform the basic tasks associated with daily living and the recovery of wrist dorsal extensor strength. Considering that all patients had severe upper limb motor dysfunction and that the observation period was short, while the recovery period of self-care ability and muscle strength was long, we believe that the experimental results are reasonable to a certain extent. In addition, we found that the combined repetitive transcranial magnetic stimulation group showed a statistically significant difference in the excitability of the motor cortex before and after treatment, whereas the combined rehabilitation machine glove group showed no statistically significant difference. We considered that this might be related to the different mechanisms of action. Perhaps the reorganization of the motor cortex caused by a short period of peripheral intervention was not reflected by cortical excitability, or perhaps there was a delay in cortical excitability? We believe further study and investigation are needed to confirm this.

As a form of central intervention, repetitive transcranial magnetic stimulation (25) mainly depends on the cerebral corpus callosum theory. The two cerebral hemispheres of the brain are connected by the corpus callosum, and the fiber bundle controls the communication of information between the two brain hemispheres, as well as how they interact with one another. When one hemisphere of the brain is stimulated, the excitability of the other hemisphere is repressed. Similarly, when a brain hemisphere is destroyed, one side of the cortex becomes less excitable while the other side becomes more excitable. Owing to prolonged cortical excitability inhibition, ipsilateral motor function recovers slowly. Repeated transcranial magnetic stimulation directly stimulates the cerebral cortex at low or high frequencies, causes neuronal polarization or depolarization, and decreases or enhances the excitability of the contralateral cortex, resulting in improved motor function recovery (26, 27). There have been numerous reports suggesting that repeated transcranial magnetic stimulation can improve the recovery of motor function when it is combined with conventional treatment (28, 29). Among them, the clinical application of low-frequency repetitive transcranial magnetic stimulation on the healthy side is more mature and extensive. According to the most recent guidelines (26), lowfrequency repetitive transcranial magnetic stimulation on the healthy side to improve hand motor dysfunction after stroke is also a strong recommendation (Grade A Recommendation). Our research also demonstrated that the repetitive transcranial magnetic stimulation is more effective than conventional treatments, and that lowfrequency stimulation of one side of the cerebral cortex can increase the excitability of the contralateral cortex, which is similar to previous findings (30). However, in our study, there was no significant correlation between the change of cortical excitability and the recovery of motor function, probably due to the small sample size, the severity of the patient's dysfunction, and the treatment time. Of course, there is also a possibility that the change of the excitatory threshold on the stimulation side may only occur due to the tolerance of the cerebral cortex to stimulation; therefore, further research is needed to explore the relationship between cortical excitability and motor function recovery.

The soft robotic glove is a peripheral rehabilitation intervention that can make the brain undergo function-related adaptive changes after focal injury by increasing the rich environment (various external stimuli, such as sensory, motor, and postural stimuli) and the repetitive skill learning of adult animals (31). It induces the development of new synapses, the growth of dendrites, and the expansion of spinous processes in the injured area of the cerebral cortex. By repeatedly stimulating the hemiplegic limb with motor and sensory input, the wearable portable soft robotic glove can increase motor control and improve motor function (32). In terms of clinical application research, Radder et al. found that whether they are used as training tools or supplementary tools, soft robotic gloves are a good choice for motor dysfunction (16). However, there is still some controversy over whether the therapeutic effect of rehabilitation machines is better than conventional equipment. One study found that robot-assisted training had similar healing effects to conventional training, with no significant additive effect (33). Our study, based on routine training combined with the peripheral intervention of soft robotic gloves, showed that patients with severe upper limb and hand dysfunction did indeed have better motor function recovery than with conventional training alone, which is consistent with the results of a 2019 study (34). We believe the controversy further suggests that the efficacy of different interventions may be related to the severity of the patient's dysfunction, the length of the disease, and the timing and duration of the interventions. And these issues are inevitable in the formulation of standardized guidelines in the field of rehabilitation therapy, and more studies are needed to further clarify them in the future.

It is possible to support the soft glove combination as a viable therapy option for people with severely impaired upper limb function after a stroke because the improved effect on motor function produced by the combination is not significantly different to that of repetitive TMS and is better than that with conventional treatment. Of course, large-scale multicenter clinical studies are still needed to further confirm whether there is a difference in the long-term effects of the two rehabilitation interventions, or whether the combination of the two can produce an effect in which one plus one is >2, which also provides a direction for our follow-up research.

## Limitations

Our study has some limitations. We only focused on the short-term effect, and did not track the long-term effect. In addition, as acupuncture treatment is highly accepted by patients in China, this study used it as a part of the routine treatment, which may not be completely consistent with the routine treatment regimen in most current studies. Furthermore, there is a concern that, despite statistical differences in several outcome indicators in this trial, the amount of change was not as significant as that in some other studies. In view of the serious degree of motor dysfunction and short treatment period of patients, we believe that the results of this study are still reasonable. Whether it has a guiding significance for clinical application is another matter and multicenter studies with large samples and prolonged treatment cycles may be needed to confirm this.

## Conclusion

For patients with severe motor dysfunction after a stroke, within a 2-week treatment period, conventional therapy combined with soft robotic gloves has the same advantages as conventional therapy combined with repetitive transcranial magnetic stimulation in terms of motor function rehabilitation, and is a safe and effective therapeutic intervention measure. For some patients, soft robotic gloves may be used as a temporary alternative to repetitive transcranial magnetic therapy if that treatment is not available.

## Data availability statement

The original contributions presented in the study are included in the article/supplementary material, further inquiries can be directed to the corresponding authors.

## Ethics statement

The studies involving human participants were reviewed and approved by Ethics Review Committee of Affiliated Hospital of Guangdong Medical University. The patients/participants provided their written informed consent to participate in this study.

## Author contributions

TW carried out clinical trial design, data analysis, and paper writing. JG and ZL conducted the feasibility analysis of the experiment, was responsible for the quality control and review of the article, and was responsible for the overall supervision, and management of the article. JT and TH was responsible for trial evaluation and data collection. All authors contributed to the article and approved the submitted version.
